# Induction of Axonal Outgrowth in Mouse Hippocampal Neurons via Bacterial Magnetosomes

**DOI:** 10.3390/ijms22084126

**Published:** 2021-04-16

**Authors:** Sara De Vincentiis, Alessandro Falconieri, Frank Mickoleit, Valentina Cappello, Dirk Schüler, Vittoria Raffa

**Affiliations:** 1 Department of Biology, University of Pisa, SS12 Abetone e Brennero 4, 56127 Pisa, Italy; sara.devincentiis@phd.unipi.it (S.D.V.); alessandro.falconieri@biologia.unipi.it (A.F.); 2 Department of Microbiology, University of Bayreuth, Universitätsstraße 30, 95447 Bayreuth, Germany; Frank.Mickoleit@uni-bayreuth.de (F.M.); Dirk.Schueler@uni-bayreuth.de (D.S.); 3 Center for Nanotechnology Innovation @NEST, Istituto Italiano di Tecnologia, Piazza San Silvestro 12, 56127 Pisa, Italy; Valentina.Cappello@iit.it

**Keywords:** stretch-growth, magnetosomes, axonal elongation

## Abstract

Magnetosomes are membrane-enclosed iron oxide crystals biosynthesized by magnetotactic bacteria. As the biomineralization of bacterial magnetosomes can be genetically controlled, they have become promising nanomaterials for bionanotechnological applications. In the present paper, we explore a novel application of magnetosomes as nanotool for manipulating axonal outgrowth via stretch-growth (SG). SG refers to the process of stimulation of axonal outgrowth through the application of mechanical forces. Thanks to their superior magnetic properties, magnetosomes have been used to magnetize mouse hippocampal neurons in order to stretch axons under the application of magnetic fields. We found that magnetosomes are avidly internalized by cells. They adhere to the cell membrane, are quickly internalized, and slowly degrade after a few days from the internalization process. Our data show that bacterial magnetosomes are more efficient than synthetic iron oxide nanoparticles in stimulating axonal outgrowth via SG.

## 1. Introduction

The mechanosensitivity of cells determines a specific response to mechanical stimulation. Mechanical stretch can modulate several different cellular functions, such as the electrical activity of cardiac muscle [1], osteogenesis [2], and the myogenic response of small arteries [3]. Although mechanosensitivity is essential to all cells, studies were mainly devoted to clarify signal mechanotransduction in those cells that play a fundamentally mechanical role. However, in the last decades, the pivotal role of mechanical force in the neuron development has been clarified and has attracted much attention. Neurons are mechanosensitive cells over three distinct ranges of force magnitude [4]. They are even more mechanosensitive than other non-neuronal cell type, by sensing, probing and responding to pico-Newton (pN) forces [5]. Recently, our team demonstrated that the generation of pN forces modulates neurite elongation, sprouting, and neuron maturation [5,6]. We developed a new methodology for stretching the axon shaft from the “inside” by developing a force of about 10 pN, which is similar or lower than those endogenously generated by the growth cone, pointing that the generation of such extremely low mechanical forces is an endogenous mechanism of axonal outgrowth [5,6]. This methodology is based on labelling the whole axon with magnetic nanoparticles (MNPs) for subsequent application of an external magnetic field gradient to induce a force generation that stretches the whole axon. Mouse hippocampal neurons were found to elongate by stretch-growth at a rate of about 6.6 ± 0.2 µm·h^−1^ without thinning or axon disconnection [5,6]. Interestingly, we found that stretch-growth occurs at a similar extent using iron oxide MNPs with different core size or different surface coatings, provided that the nanoparticle concentration and the labelling process is optimized for the generation of a force in the range of 10 pN in the axon shaft [5]. MNPs are exceptional nanotools for studying the signal mechanotransduction during stretch-growth [4], with a view for future translational therapies [5]. Static magnetic fields are extensively used in medicine and several magnetic nanoparticles are approved for human use [7] that allows to speculate that this methodology has a pre-clinical and clinical potential for treating nerve injuries or neurodegenerative disorders. In the attempt to optimize protocols for future pre-clinical studies, our attention was attracted by an approach of green chemistry to synthetize a new type of MNPs, namely, magnetosomes.

Magnetosomes are organelles that enable magnetotactic bacteria (MTB) to align along geomagnetic field lines (magnetotaxis) [8]. In the MTB model organism *Magnetospirillum gryphiswaldense*, they consist of a cuboctahedral core of chemically pure magnetite (Fe_3_O_4_) that is naturally enveloped by a biological membrane [9,10]. The latter consists of phospholipids and a set of more than 30 different magnetosome-specific proteins [11,12,13,14] that fulfil essential functions in magnetosome biosynthesis (i.e., vesicle formation by invagination of the cytoplasmic membrane, nucleation, magnetite crystal growth, and chain-like arrangement of the particles within the cells [15]. Precise control on each step of biomineralization generates single magnetic domain core–shell nanoparticles with a strong magnetization and a stable magnetic moment at physiological temperatures. Furthermore, magnetosomes exhibit a high crystallinity, uniform shape, and a narrow size distribution (on average ~35 nm in diameter), which can hardly be achieved by chemical synthesis [16,17,18,19].

Magnetosomes can be isolated from the cells and purified with the intact membrane, thereby ensuring that the particles are colloidal stable and dispersible [13,14]. In addition, the enveloping membrane is accessible for chemical coupling [20,21,22] or in vivo functionalization by genetic engineering [23]. Previous approaches utilized highly abundant magnetosome membrane proteins as anchor molecules for the display of fluorophores, enzymes, or coupling groups such as antibodies or molecular connectors [24,25,26,27,28,29]. Based on these properties, isolated magnetosomes were already successfully tested in magnetic hyperthermia, as contrast agents for magnetic imaging techniques, as carriers for controlled drug delivery or in cell separation assays [30,31,32,33,34]. Furthermore, the particles were shown to differentially interact with mammalian cells (depending on the cell line investigated), ranging from cell surface reactions to particle internalization [35,36,37]. Interestingly, potential applications of magnetic manipulation of magnetosomes in mammalian cell lines were also investigated. In the work of Shin and colleagues [38], cellular growth of HeLa cells has been modulated by the communication between internalized magnetosomes and exposure to an external magnetic field. The synergistic interaction of magnetism furthermore induced anti-apoptotic effects and significantly increased cellular growth. Similarly, in [35], U87MG cells were exposed to magnetosomes, and an increase in optical absorbance was detected after 24 h of incubation, which might be explained by either an increase in cell viability or proliferation induced by magnetosome treatment. Thus, the application of magnetosomes as theranostic agents might also offer a promising strategy for targeted cell therapy with controllable cell viability. Besides unspecific magnetic “labelling” of the cell surface, more specific interactions have also been envisioned by magnetosome display of immuno-stimulatory ligands and cell recognition elements [39,40,41,42]. For instance, the expression of CD40L (a ligand functional in immune responses) on the magnetosome surface enabled specific binding to CD40-decorated sensor cells, thereby triggering signaling cascades upon magnetosome binding [39]. Magnetosomes might therefore represent versatile “tools” for the stimulation of eukaryotic cells.

In our study, magnetosomes isolated from *M. gryphiswaldense* were shown to possess distinctive abilities to induce stretch-growth of mouse hippocampal neurons. Thereby, a strong cellular interaction was observed even at very low concentrations.

## 2. Results and Discussion

### 2.1. Magnetosome Cell Interactions

The cytotoxicity/biocompatibility of bacterial magnetosomes has been tested on various mammalian cell lines, including primary cells as well as cancer cells [14,37,43,44,45]. Although the tested cell lines differed in their sensitivity to magnetosome treatment, viability values suggested biocompatibility for a broad concentration range. Thus, even for relatively high particle amounts (up to 400 µg Fe mL^−1^), cell viability was only slightly affected, even for more sensitive primary cells [14,37].

**Figure 1:**
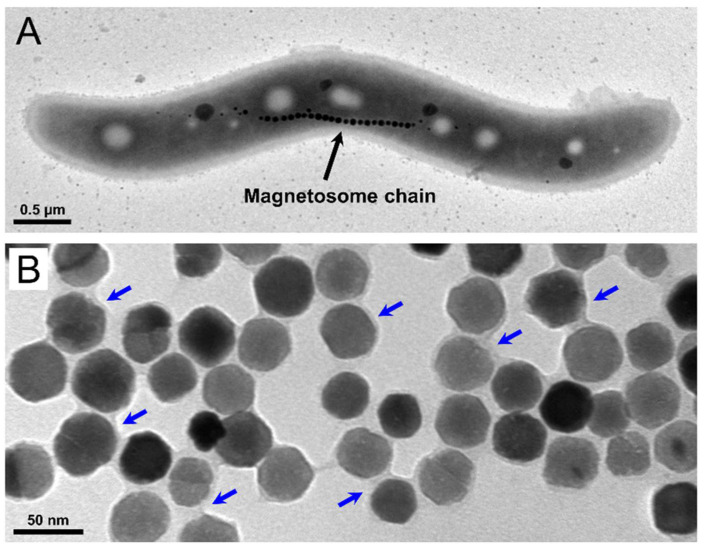
Magnetosomes in *M. gryphiswaldense*. (**A**) TEM micrograph of a wild-type cell of *M. gryphiswaldense*. The latter biomineralizes up to 40 magnetosomes, arranged in a chain-like manner at midcell. (**B**) TEM image of a suspension of isolated/purified magnetosomes (negatively stained), containing well-dispersed particles. Magnetosomes consist of a magnetite core that is surrounded by a biological membrane (indicated by arrows). The latter consists of phospholipids and a set of magnetosome-specific proteins, and provides colloidal stability.

**Figure 2:**
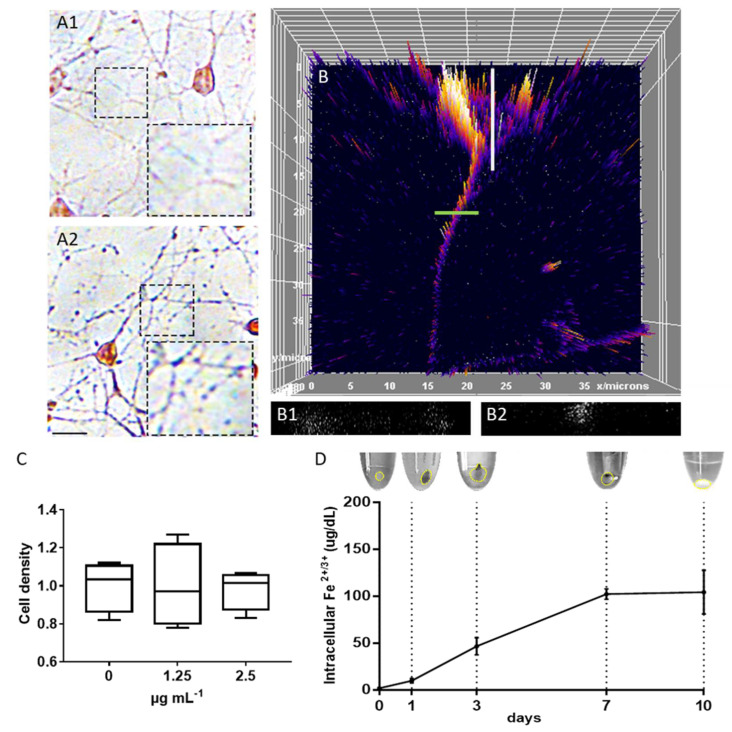
Imaging of cells without (**A1**) and with magnetosomes (**A2**) stained with iron stain kit, scale bar: 20 µm. (**B**) 3D surface plot, top view. Re-slice of cell soma (white line) shows no particles in the nucleus (**B1**). Re-slice of axon (green line) shows a uniform distribution of fluorescent puncta (**B2**). (**C**) Dose–response assay: the number of neurons was counted at day in vitro (DIV) 3; *n* = 4 (box plot, min-to-max); *n* = 4; one-way ANOVA; *p* = 0.98, F = 0.01759. (**D**) Intracellular levels of Fe^2+/3+^ versus time before (0 time point) and after magnetosome treatment and relative pellets collected after cell lysis; *n* = 3.

In this study, magnetosomes from *M. gryphiswaldense* were isolated and purified with their intact biological membrane (Figure 1), exhibiting an overall particle size of 39.7 ± 8.1 nm, a hydrodynamic diameter of 51.2 ± 7.2 nm, and high saturation magnetization (≥70 Am^2^ kg^−1^) [46,47]. Magnetosomes were tested on mouse hippocampal neurons to exclude any toxicity and data confirm that the particles can be safely administered to cells to the low concentrations (<5 µg Fe mL^−1^) (Figure 2C) required for stretch-growth.

Next, we studied the interaction of magnetosomes with neurons, focusing on the intracellular localization and particle degradation, which is relevant for the estimation of the mechanical force generated into the axonal shaft. Cells treated with magnetosomes appeared morphologically similar to the control, and magnetosomes were visible as single spots into the axons after Prussian Blue staining (without magnetosomes, Figure 2A1, and with 2.5 µg mL^−1^ magnetosomes, Figure 2A2). In order to better characterize the intracellular localization of these particles into the neurons, we used fluorescently labelled magnetosomes (Figure 2B). Fluorescence was easily detected in the cytoplasm of both cell soma and neurites. By analyzing the reconstruction of neuron volume via Z-stack re-slice, magnetosomes as small puncta were detected intracellularly. In the neuronal soma, we observed absence of particles in the nucleus (Figure 2B1), but they were present in the cytoplasm, as well as in the axon (Figure 2B2). This result is in line with previous observations that we collected with iron oxide magnetic nanoparticles in PC12 cells and hippocampal neurons [5,6]. In order to characterize the intracellular degradation dynamics, we performed an incubation with magnetosomes for 4 h, followed by an extensive washing step. Then, we collected cell pellets at different time points (1, 3, 7, and 10 day post-treatment, dpt). For each time point, cell pellets were lysed, the supernatant was collected and separated by centrifugation from cell membrane fragments for measuring the intracellular levels of Fe^2+^ and Fe^3+^ that is mark of particle dissolution. Collected data point that magnetosomes are mainly intact at 1 dpt, as documented by the brown-stained pellet and the low intracellular level of Fe ions (Figure 2D). From 3 dpt, magnetosomes start to disappear from the pellet and the intracellular iron concentration increases, consistent with the assumption that magnetosomes inside neurons are slowly dissolving. Starting from 7 dpt, the Fe intracellular concentration level stabilizes and the particles totally disappear from the cell membrane pellets, suggesting the process of intracellular magnetosome degradation is now sustained. These data are also consistent with observations previously collected with hippocampal neuron treated with different magnetic nanoparticles [5].

### 2.2. Magnetosomes Induce Stretch-Growth

Recently, we demonstrated in PC12 cell line and hippocampal neurons that neurite labelling with superparamagnetic iron oxide nanoparticles (SPIONs) enables them to be stretched through the dragging force created by an external magnetic field [5,6]. In this study, we tested the capability of magnetosomes to obtain the same effect, in terms of induction of stretch-growth. 

**Figure 3:**
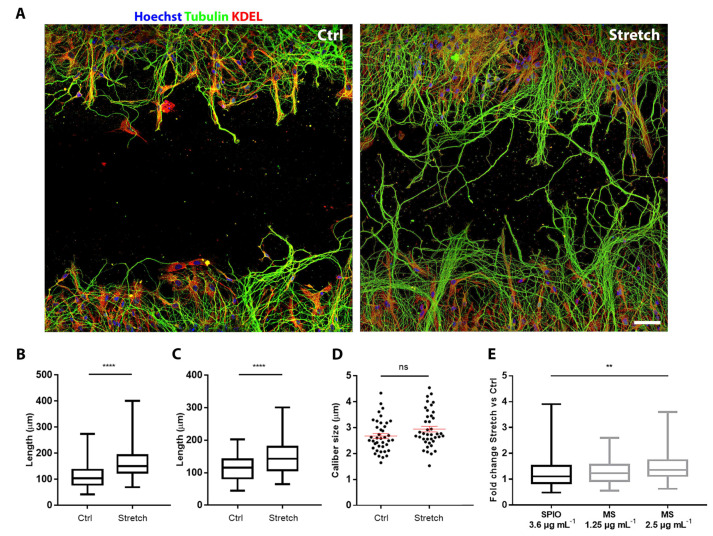
Stretch-growth assay. (**A**) Length of primary axons in control (left) and stretched condition (right), scale bar: 50 µm. (**B**) Length of primary axons of neurons treated with 1.25 µg Fe mL^−1^ of magnetosomes (box plot, min-to-max); *n* = 120 from four independent assays. *T*-test for unpaired data, *p* < 0.0001, t = 5.881, df = 238. (**C**) Length of primary axons of neurons treated with 2.5 µg Fe mL^−1^ of magnetosomes (box plot, min-to-max); *n* = 120 from four independent assays. *T*-test for unpaired data, *p* < 0.0001, t = 8.152, df = 238. (**D**) Axon caliber of neurons treated with 2.5 µg mL^−1^ of magnetosomes (scatter dot plot, mean ± SEM). *T*-test for unpaired data, *n* = 40 from four independent assays, *p* = 0.07, t = 1.817, df = 78. (**E**) Comparison between fold change of axon length in stretched versus non-stretched conditions of samples treated with 3.6 µg Fe mL^−1^ of SPIONs, with 1.25 µg mL^−1^ of magnetosomes (MS) and 2.5 µg mL^−1^ MS (box plot, min-to-max); one-way ANOVA test, *n* = 120 neurons from four independent assays, *p* = 0.021.

The elongation analysis was carried out on cells labelled 4 h after plating with magnetosomes and exposed from day in vitro (DIV) 1 to DIV3 to the magnetic field (hereafter, labelled as stretch group) or a null magnetic field (labelled as control group). Figure 3 shows hippocampal neurons cultured on two distinct areas separated by a 0.5 mm cell-free gap region. A comparison between control and stretched condition reveals the dramatic effect of the stretch on the length of axons after 48 h of continuous stretching. For the quantification of this increase, we tested the two doses for which we excluded any toxicity on neurons (1.25 and 2.5 µg mL^−1^). Each experiment was repeated four times and analyzed by two different operators. In each experiment and with each dose tested, we found a statistically highly significant increase (*p* < 0.0001) in axonal length in comparison to the control groups (Figure 3B,C). To demonstrate that the observed length increase was real growth and not a viscoelastic deformation, the average thickness of neurites treated with 2.5 µg mL^−1^ of magnetosomes was calculated (Figure 3D). In line with our previous findings [5,6], no differences in size of neurites were found (*p* = 0.07).

**Figure 4:**
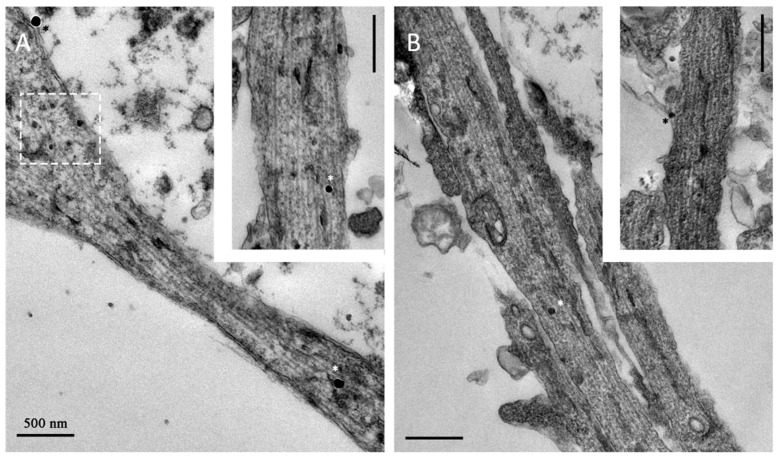
TEM analysis of hippocampal neurons incubated for 72 h with 1.25 µg mL^−1^ of magnetosomes (**A**) and with 3.6 µg Fe mL^−1^ of superparamagnetic iron oxide nanoparticles (SPIONs) (**B**). Representative micrograph of axon cross-sections. White and black stars highlight some particles in the axoplasm and at the membrane, respectively. Scale bar: 500 nm.

Additionally, we made a comparison between the effect induced by biosynthesized magnetosomes and synthetic iron oxide nanoparticles. In previous works, we tested nanoparticles with different size (iron oxide core from 2 to 80 nm), magnetic properties, and surface coatings. Here, for comparison, we selected the commercially available SPIONs (core size 75 ± 10 nm, saturation magnetization 59 Am^2^kg^−1^), i.e., the most powerful inducer of stretch-growth that have been tested so far from our team [5,6]. Figure 3E shows the elongation rate, given as fold change of the stretch group versus the control group. Specifically, the stretch-growth induced an increase in the elongation rate of 1.43, 1.58, and 2.08 µm h^−1^, using respectively, 3.6 µg Fe mL^−1^ of SPIONs, and 1.25 and 2.5 µg Fe mL^−1^ of magnetosomes. Surprisingly, the treatment with 2.5 µg Fe mL^−1^ of magnetosomes provided a statistically significant increase in the length of neurites compared to samples treated with SPIONs 3.6 µg Fe mL^−1^ (*p* = 0.021). We were wondering if this increase could be associated to differences in the cell–particles interactions between the two groups. The study of the axonal ultrastructure shows that magnetosomes adhere to the cell membrane of developing axons and, after internalization, localize as single particles within the axon (Figure 4A). Similarly, SPIONs are localized as single spots to both the cell membrane and axoplasm of hippocampal neurons (Figure 4B), suggesting an analogous internalization pathway and localization pattern between the two groups. However, axons treated with magnetosomes show a very high density of smaller less electron-dense spots whose size is compatible with the mean size of the magnetosome iron oxide core (Figure 4A, dashed white rectangle) that could account for a strong ability of magnetosomes to enter the axons.

Considering the smaller size of the inorganic core and the lower Fe concentration used for cell labelling (Table 1), we can conclude that the superior magnetic behavior of the biosynthesized magnetosomes (Table 1), together with their strong ability to accumulate inside the axons, makes them a superior nanotool for inducing stretch-growth of hippocampal neurons than artificial SPIONs.

## 3. Materials and Methods

### 3.1. Animals

Animal procedures were performed in strict compliance with protocols approved by the Italian Ministry of Public Health (MoH) and of the local Ethical Committee of University of Pisa, in conformity with the Directive 2010/63/EU (project license approved by the MoH on 22 November 2018). C57BL/6J mice (post-natal P0-P1) were used. They were kept in a regulated environment (23 ± 1 °C, 50 ± 5% humidity) with a 12 h light-dark cycle with food and water ad libitum.

### 3.2. Cell Culture

The dissection of both hippocampi of P0–P1 mice of both sexes was conducted in a solution of D-glucose 6.5 mg mL^−1^ (Sigma, Darmstadt, Germany) in DPBS (Invitrogen, Carlsbad, CA, USA); chemical digestion and mechanical dissociation were performed to isolate hippocampal neurons. Cells were seeded in high-glucose DMEM (Invitrogen, Carlsbad, CA, USA) with 10% fetal bovine serum (FBS; Invitrogen, Carlsbad, CA, USA), 100 IU mL^−1^ penicillin (Invitrogen, Carlsbad, CA, USA), 100 μg mL^−1^ streptomycin (Invitrogen, Carlsbad, CA, USA), and 2 mM GlutaMAX (Invitrogen; Carlsbad, CA, USA) on poly-D-lysine (PDL; Sigma, Darmstadt, Germany)-coated surfaces at a density of 160 cells mm^−2^. Cells were incubated at 37 °C in a saturated humidity atmosphere containing 95% air and 5% CO_2_. After 4 h, the medium was replaced with Neurobasal-A medium (Invitrogen; Carlsbad, CA, USA) modified with B27 (Invitrogen; Carlsbad, CA, USA), 2 mm GlutaMAX, 50 IU mL^−1^ penicillin, and 50 μg mL^−1^ streptomycin.

### 3.3. Chemically Synthesized Nanoparticles

SPIONs, commercially available (Fluid-MAG-ARA, Chemicell; Berlin, Germany), have an inorganic core of iron oxide, ~75 ± 10 nm in diameter, and saturation magnetization of 59 Am^2^kg^−1^, as stated from the supplier. They have an organic shell preventing aggregation, made of glucuronic acid, and the hydrodynamic diameter is 100 nm.

### 3.4. Cultivation of Magnetospirillum gryphiswaldense

The wild-type (WT) strain of *M. gryphiswaldense* MSR-1 [48,49] was grown in modified flask standard medium (FSM; 10 mM 4-(2-hydroxyethyl)-1-piperazineethanesulfonic acid (HEPES), 15 mM potassium lactate, 4 mM NaNO_3_, 0.74 mM KH_2_PO_4_, 0.6 mM MgSO_4_, 50 μM Fe^3+^-citrate, 3 g L^−1^ soy peptone, and 0.1 g L^−1^ yeast extract, at pH 7.0) as previously described [50]. Cultivation was performed in 5 L flasks under moderate shaking (120 rpm) at 28 °C. For the formation of magnetite, a headspace-to-liquid ratio of ~1:4 with air in the headspace was applied. Under these conditions, the oxygen concentration in the medium declined with increasing cell numbers, thereby reaching microoxic conditions and inducing magnetosome biomineralization [51]. Cells were harvested in the late exponential phase by centrifugation (9000× *g*, 4 °C, 20 min). The cell pellets were subsequently washed with 20 mM HEPES and 5 mM ethylenediaminetetraacetate (EDTA), at pH 7.2, and stored at −20 °C until further use.

Chemicals were purchased from Merck (Darmstadt, Germany) or Carl Roth (Karlsruhe, Germany) with purity grades ≥98%.

### 3.5. Magnetosome Isolation and Purification

Magnetosomes were isolated and purified as previously described [13,14]. Cell pellets of *M. gryphiswaldense* were resuspended in 50 mM HEPES, 1 mM EDTA, at pH 7.2, and cell disruption was performed by passing the suspension 3–5 times through a microfluidizer system (M-110 L, Microfluidics Corp., Westwood, MA, USA) equipped with a H10Z interaction chamber at 124 MPa. Afterwards, the crude extract was passed through a MACS magnetic-separation column (5 mL; Miltenyi, Bergisch Gladbach, Germany) placed between two neodymium-iron-boron magnets (each 1.3 T). Thereby, the magnetosomes were retained within the column, whereas non-magnetic cellular compounds passed the column and were instantly eluted. The column was subsequently washed with 50 mL 10 mM HEPES, 1 mM EDTA, at pH 7.2, followed by 50 mL high-salt buffer (10 mM HEPES, 1 mM EDTA, and 150 mM NaCl, at pH 7.2) to remove electrostatically bound impurities, and again by 50 mL 10 mM HEPES and 1 mM EDTA, at pH 7.2. The magnets were finally removed, and the magnetosomes were eluted with ddH_2_O. As further purification step, the particle suspension was centrifuged through a 60% *w*/*v* sucrose cushion (in 10 mM HEPES and 1 mM EDTA, at pH 7.2) for 2 h at 200,000× *g* and 4 °C. During centrifugation, the magnetosomes formed a pellet at the bottom of the tube (due to their high density), whereas residual impurities were retained in the cushion. Lastly, the particles were resuspended in 10 mM HEPES, at pH 7.0, and stored in Hungate tubes at 4 °C under a nitrogen atmosphere.

### 3.6. Determination of Iron Concentrations

The iron content of suspension of isolated magnetosomes was determined by atomic absorption spectroscopy (AAS). For this, 25–50 µL of magnetosome suspension was mixed with 69% nitric acid (final volume 3 mL) and incubated for 3 h at 98 °C. Samples were subsequently filled up to a volume of 3 mL with ddH_2_O and analyzed using an Analytik Jena contrAA 300 high-resolution atomic absorption spectrometer (Analytik Jena, Jena, Germany) equipped with a 300 W xenon short-arc lamp (XBO 301, GLE, Berlin, Germany) as continuum radiation source. The equipment presented a compact high-resolution double monochromator (consisting of a prism pre-monochromator and an echelle grating monochromator) and a charge-coupled device (CCD) array detector with a resolution of about 2 pm per pixel in the far ultraviolet range. Measurements were performed at a wavelength of 248.3 nm using an oxidizing air/acetylene flame. The number of pixels of the array detector used for detection was 3 (central pixel 1). All measurements were carried out in quintuplicates (*n* = 5), each as a mean of three technical replicates.

### 3.7. Fluorescent Labelling of Isolated Magnetosomes

Magnetosomes were fluorescent-labelled by reaction with DyLight 488 Amine-Reactive Dye (NHS ester-activated derivative of high-performance DyLight 488; Thermo Scientific, Waltham, MA, USA). Thereby, the labelled particles were shown to exhibit an up to 12-fold increased fluorescence compared to EGFP magnetosomes (genetically engineered magnetosomes that display up to 200 EGFP molecules on the surface) [37].

Briefly, 1.5 µg of the fluorescent dye (stored as a 10 mg mL^−1^ stock solution in dimethylformamide) was added to 1 µg magnetosomes (in 50 mM NaHCO_3_, pH 9.0) and incubated in the dark for 2 h at 16 °C. Excess dye was removed by extensive washing in which the particles were several times pelleted by centrifugation (4000× *g*, 4 °C, 30 min) and, after discarding the supernatant, resuspended in 10 mM HEPES, at pH 7.0. Success of the labelling reaction was confirmed by fluorescence measurements (535 nm) using an Infinite M200pro plate reader (Tecan, Crailsheim, Germany).

### 3.8. Sterile Filtration of Magnetosome Suspensions

For application under sterile conditions, magnetosomes were sterile filtrated as described previously [39]. Suspensions of WT particles or DyLight 488—labelled magnetosomes were diluted with 10 mM HEPES, at pH 7.0, to an Fe concentration of ~50 µg mL^−1^ and filtrated using a 0.22 μm PVDF sterile filter (Roth, Karlsruhe, Germany). The particles were collected by low-spin centrifugation (4000× *g*, 4 °C, 30 min) and resuspended in a small volume of ddH_2_O. After determination of the Fe content by AAS, the suspensions were diluted to a final Fe concentration of 500 µg mL^−1^. 

### 3.9. Transmission Electron Microscopy

For transmission electron microscopy (TEM) analyses of *M. gryphiswaldense* cells and isolated magnetosomes, specimens were deposited onto carbon-coated copper-mesh grids (Science Services, Munich, Germany). Isolated particles were additionally negatively stained with 2% uranyl acetate. TEM was performed on a JEOL 1400 (JEOL, Tokyo, Japan) with an acceleration voltage of 80 kV. Images were taken with a Gatan Erlangshen ES500W CCD camera.

For localization studies, neurons, both control and stretched, were fixed with 1.5% glutaraldehyde in 0.1 M sodium cacodylate buffer, at pH 7.4. After rinses with the same buffer, cells were post-fixed in reduced osmium tetroxide solution (1% OsO_4_, 1% K_3_Fe(CN)_6_, and 0.1 M sodium cacodylate buffer), stained with our homemade staining solution [52], dehydrated in a growing series of ethanol, and flat-embedded in epoxy resin. 

Ultra-thin sections (90 nm) were cut with a UC7 LEICA ultramicrotome (UC7—Leica Microsystems, Vienna, Austria) and collected on 300 mesh copper grids (EMS—Electron Microscope Science, Hatfield, PA, USA).

Images for morphological characterization have been collected with a Transmission electron microscope Zeiss LIBRA 120 Plus operating at 120 KeV equipped with an in-column omega filter.

### 3.10. Analytical Methods

The optical density (OD) and magnetic response (C_mag_) of *M. gryphiswaldense* cultures were monitored by photometric measurements at 565 nm as reported previously [53]. Thereby, the OD_565_ directly correlates with the cellular growth. C_mag_ represents a light-scattering based proxy for the average magnetic orientation of bacterial cells and relates to the average magnetosome numbers in cell populations, allowing semiquantitative estimations of the particle content.

Hydrodynamic diameters of isolated magnetosomes were determined with a Malvern Zetasizer Nano-ZS (Malvern Panalytical, Malvern, UK). Measurements were performed in the automatic mode (wavelength 638 nm) at 25 °C on highly diluted particle suspensions using DTS1070 cuvettes.

### 3.11. Bio-Synthetized versus Artificially Synthetized Nanoparticles

The summary of the main properties of bacterial magnetosomes and a comparison with the commercial SPIONs used in this work is provided in Table 1. As for the magnetic properties, SPIONs are multi-domain superparamagnetic nanoparticles, as stated from the supplier. Recent studies on the magnetic properties of isolated magnetosomes revealed the co-existence of stable single domain (SSD) and superparamagnetic particles (SP) [33,54,55]. Although the particles exhibit a narrow size distribution, the suspensions are polydisperse to some extent [14]. Suspensions of wild-type-like sized magnetosomes also contain SP particles, however, the SSD particles dominate [33].

### 3.12. Magnetic Field

Experiments were conducted in 35 mm Petri dishes placed inside a Halbach-like cylinder magnetic applicator, which provided a constant magnetic field gradient of 46.5 Tm^−1^ in the radial centrifugal direction [5,6,56].

### 3.13. Stretching Assay

For stretching assay 80,000 cells were seeded on a 14 mm glass coverslip. Alternatively, seeding of hippocampal neurons was performed in a 2-well silicone insert (80209; IBIDI, Gräfelfing, Germany), at a density of 100,000 cells per well. After letting the neurons attach for 4 h, the silicone insert was removed in order to generate a cell-free gap region of 500 µm. For both supports used, particles were added 4 h after seeding (DIV0.17). At DIV1, the culture was placed in the magnetic applicator (Stretch group) or outside (Control group). At DIV3, samples were fixed and stained for immunofluorescence.

### 3.14. Toxicity Test

Toxicity of magnetosomes was evaluated by performing dose–response assays. At DIV3, cells were fixed and stained for immunofluorescence and the number of viable neurons was counted.

After lysis, cell membranes were precipitated from the lysate by centrifugation (18,000 rpm, 5 min). An iron assay kit (DIFE-250, QuantiChrom) was performed on cell lysate for intracellular iron quantification, and the absorbance was measured at a wavelength of 590 nm.

### 3.15. Particle Localization

An iron stain kit (HT20-1KT, Sigma) was applied for particle localization. In addition, fluorescent magnetosomes were used. 

### 3.16. Immunostaining

For the immunostaining performed for stretching and toxicity analysis, samples were fixed in 2% PFA, permeabilized with 0.5% Triton X-100, and blocked with 5% FBS at room temperature. Primary antibody TUBB3 (#T8578, 1:500, Sigma, Darmstadt, Germany) was diluted in 3% FBS/0.2% Triton X-100 in DPBS and incubated overnight at 4 °C. Samples were then washed and incubated with secondary antibody (R6393, 1:500, Invitrogen, Carlsbad, CA, USA) and Hoechst 33342 (H3570, 1:1000, Invitrogen, Carlsbad, CA, USA). All images were acquired using a fluorescent microscope (TE2000-U, Nikon, Minato, Tokyo, Japan).

For the immunostaining performed on 2-well silicone insert, cells were fixed in 2% PFA and 7.5% sucrose, permeabilize in 0.1% Triton X-100 and blocked with 5% goat serum (GS) at room temperature. Primary antibodies TUBB3 (#T8578, 1:500, Sigma, Darmstadt, Germany) and KDEL (PA1-013, 1:200, Thermo Scientific, Waltham, MA, USA) were incubated overnight at 4 °C. Samples were then washed and incubated with secondary antibody (#A21449 and #06380, 1:500, Thermo Scientific, Waltham, MA, USA) Hoechst 33342 (H3570, 1:1000, Invitrogen, Carlsbad, CA, USA). All images were acquired using a laser scanning confocal microscope (Eclipse Ti, Nikon, Minato, Tokyo, Japan).

### 3.17. Image Analysis

The analysis of the elongation was performed using image analysis software ImageJ. Neurite length *l* was evaluated using the plugin NeuronJ [57], and 120 non-interconnected primary axons were analyzed from 10× magnification images (randomly acquired).

For axon thickness, a population of 40 axons was analyzed from 10× magnification images (randomly acquired). For each axon, the longest path *l* has been considered, and the thickness *s* was calculated as *s* = *A*/*l*, *A* being the axon area related to that path. Area was calculated from images after threshold normalization, binary conversion.

### 3.18. Statistical Analysis

Data were plotted and analyzed with GraphPad software, version 6.0. Significance was set at *p* ≤ 0.05. Statistical power analyses have been performed with G-power software.

## 4. Conclusions

Neurons are mechanosensitive cells, and the application of extremely low force positively modulate axonal elongation, neurite branching, and neuron maturation [5,58,59,60,61,62,63,64,65]. Many studies demonstrated that magnetic nanoparticles are an efficient tool for magnetizing axons, and the subsequent application of a magnetic field gradient can generate such extremely low forces that drive productive axonal outgrowth [5,6,56,66,67]. The efficiency of this stretching protocol depends on the ability of the nanoparticles to label axons and on their magnetic behavior. In this study, we demonstrate that superior magnetic properties of bacterial magnetosomes together with the strong interactions with neurons, probably facilitated by their membranous structure, account for their extraordinary ability to induce stretch-growth. This finding would expand the range of biotechnological applications of bacterial magnetosomes also to the neuroscience field, which has been poorly explored to date.

## Figures and Tables

**Table 1:** Comparison between superparamagnetic iron oxide nanoparticles (SPIONs) and bacterial magnetosomes.

	SPIONs	Magnetosomes
*Core diameter*	75 ± 10 nm	39.7 ± 8.1 nm
*Hydrodynamic diameter*	100 nm	51.2 ± 7.2 nm
*Saturation magnetization*	59 Am^2^ kg^−1^	70–90 Am^2^ kg^−1^
*Coating*	Glucuronic acid	Proteinaceous phospholipid membrane
*Concentration for cell labelling*	3.6 µg Fe mL^−1^	1.25–2.5 µg Fe mL^−1^

## Data Availability

Data available on request due to restrictions eg privacy or ethical.
